# Impact of Surgical Timing on Fracture Healing in Tibial Shaft Injuries: A Comparative Review of Intramedullary Nailing Techniques

**DOI:** 10.7759/cureus.70978

**Published:** 2024-10-07

**Authors:** Samarth Kashyap, Ratnakar Ambade, Suhas Landge, Ankur Salwan

**Affiliations:** 1 Orthopedics and Traumatology, Jawaharlal Nehru Medical College, Datta Meghe Institute of Higher Education & Research, Wardha, IND

**Keywords:** delayed surgery, early intervention, fracture healing, intramedullary nailing, surgical timing, tibial shaft fractures

## Abstract

Tibial shaft fractures are a prevalent and challenging orthopedic injury, often resulting from high-energy trauma. Optimal management of these fractures is crucial to prevent complications such as nonunion, malunion, and prolonged functional impairment. Intramedullary nailing (IMN) is widely regarded as the gold standard for treating these injuries due to its ability to provide stable fixation, preserve soft tissues, and enable early mobilization. However, the timing of surgical intervention remains a topic of ongoing debate. Early surgical intervention, defined as surgery within 24-48 hours of injury, is advocated for its potential to reduce hospital stay, facilitate early mobilization, and decrease the risk of secondary complications such as compartment syndrome. Conversely, delayed intervention allows for patient stabilization and reduction of soft tissue swelling, potentially lowering the risk of infection and other complications. This comprehensive review aims to evaluate the impact of surgical timing on fracture healing outcomes in tibial shaft fractures treated with IMN. It explores the benefits and limitations of early versus delayed surgery, emphasizing their influence on union rates, healing time, and complication rates. Furthermore, the review examines different IMN techniques, including reamed versus unreamed and static versus dynamic nailing, to determine their role in optimizing fracture healing. By synthesizing current evidence, this review provides critical insights into the optimal timing and technique for IMN in tibial shaft fractures, ultimately aiming to guide clinical decision-making and improve patient outcomes. Future research should focus on randomized controlled trials to establish standardized surgical timing and technique selection guidelines in this patient population.

## Introduction and background

Tibial shaft fractures are among the most prevalent long bone fractures, representing approximately 15% of adult fractures [1]. These injuries frequently result from high-energy trauma, such as motor vehicle accidents, sports-related incidents, and falls from significant heights, making them a considerable concern in both orthopedic and trauma care settings. The unique anatomical position of the tibia, being largely subcutaneous along most of its length, predisposes it not only to fractures but also to complications such as open wounds, soft tissue damage, and compromised vascular supply [2]. The implications of tibial shaft fractures extend beyond the immediate injury, often leading to prolonged morbidity due to issues like delayed union, nonunion, or malunion. These complications can significantly hinder a patient’s functional recovery and overall quality of life. This underscores the need for timely and effective management strategies that ensure proper alignment and stability to facilitate optimal fracture healing [3]. Achieving optimal fracture healing is crucial for restoring limb function, particularly in the case of the tibia, which plays a vital role in weight-bearing and locomotion. Inadequate or improper healing of tibial shaft fractures can lead to chronic pain, instability, and long-term complications such as post-traumatic arthritis, especially if joint congruity is not maintained [4]. These outcomes affect the patient’s physical capabilities and have a significant socio-economic impact due to the extended recovery periods and rehabilitation required. Therefore, it is imperative to establish effective treatment protocols that promote fracture healing and minimize the risk of complications, ensuring a successful return to pre-injury functional levels [5].

Surgical timing plays a critical role in the management of tibial shaft fractures and has the potential to significantly influence the course of fracture healing. Early surgical intervention, typically performed within 24-48 hours post-injury, is often recommended for its potential benefits, including reduced hospital stays, facilitated early mobilization, and the prevention of secondary complications such as compartment syndrome [6]. However, early surgery is not without its risks, such as increased chances of infection and additional soft tissue damage, particularly in cases of open fractures. On the other hand, delayed surgical intervention stabilizes the patient’s overall condition and resolves soft tissue swelling, potentially reducing complications and improving surgical outcomes. Thus, the timing of surgery must be carefully considered, balancing the benefits and risks to optimize patient outcomes and fracture healing [7]. Intramedullary nailing (IMN) has become the preferred treatment method for tibial shaft fractures due to its biomechanical advantages and minimally invasive nature. This technique provides strong stabilization, preserves the fracture hematoma, and enables early weight-bearing, which are critical factors in promoting effective fracture healing. Despite these benefits, there is ongoing debate regarding the choice of nailing techniques, such as reamed versus unreamed or static versus dynamic nailing, and the optimal timing for performing the procedure [8]. The variations in these approaches can significantly impact clinical outcomes, making establishing evidence-based guidelines for their use essential. This review aims to provide a comprehensive analysis of the impact of surgical timing on the outcomes of different IMN techniques, highlighting the need for a clear understanding of these factors to inform clinical practice and improve patient care [9].

The primary objective of this review is to compare the impact of early versus delayed surgical intervention on fracture healing in tibial shaft injuries. By systematically reviewing the current evidence, this article seeks to elucidate the benefits and drawbacks of each approach in terms of union rates, complications, and overall functional outcomes. Additionally, the review aims to evaluate the various IMN techniques employed in managing tibial shaft fractures, analyzing their respective outcomes and effectiveness. Through this analysis, the review will offer insights into the optimal surgical timing and technique selection, providing clinicians with evidence-based recommendations to enhance patient outcomes in treating tibial shaft fractures.

## Review

Anatomy and biomechanics of tibial shaft

The tibial shaft is the long, triangular segment of the tibia situated between the proximal and distal ends. It is characterized by three distinct surfaces (posterior, medial, and lateral) and three borders (anterior, interosseous, and medial). The anterior border is the most prominent and easily palpable, extending from the tibial tuberosity to the distal portion of the bone. The interosseous border connects to the fibula via the interosseous membrane, while the medial border is most pronounced along the medial aspect of the middle third of the tibia [10]. The tibial shaft has a robust blood supply primarily from the nutrient artery, which enters the bone proximal to the soleal line on the posterior surface. Additional blood flow is provided by the anterior and posterior tibial arteries [11]. Fracture healing in the tibial shaft progresses through the same general phases as other long bones. The process begins with the inflammatory phase, where bleeding at the fracture site leads to hematoma formation and initiates an inflammatory response. This is followed by the soft callus phase, during which fibroblasts and chondrocytes proliferate to form a fibrocartilage bridge across the fracture gap. Next, the hard callus phase ensues, replacing the soft callus with a hard callus composed of woven and lamellar bone, stabilizing the fracture. Finally, the remodeling phase restores the bone’s original shape and strength [12]. Several mechanical and biological factors influence the healing of tibial shaft fractures. Rigid fixation using intramedullary nails or plates promotes primary bone healing, while external fixation or casting, which provides relative stability, leads to secondary bone healing through callus formation [13]. Healing can be compromised by extensive soft tissue damage, devascularization, or large bone defects caused by trauma or infection. Comminuted fractures, characterized by multiple bone fragments, present additional challenges in stabilization and healing. Systemic factors such as diabetes, smoking, and malnutrition further hinder the healing process [13].

IMN techniques

IMN has a rich history dating back to the early 20th century. The technique was pioneered by Gerhard Küntscher in 1939 during World War II when he used a V-shaped stainless-steel nail for femoral fractures. This innovation significantly improved recovery times for injured soldiers [14]. Over the following decades, IM nail designs evolved, with the cloverleaf-shaped nail introduced in the late 1940s. This new design provided better rotational stability and reduced complication rates. Additional advancements, such as reaming techniques and locking mechanisms, further improved the efficacy and safety of IMN, making it a preferred method for treating various types of fractures [14]. IMN is primarily indicated for tibial shaft fractures, especially diaphyseal and extra-articular fractures. It suits various fracture types, including spiral and comminuted fractures [15]. The technique is favored for its minimally invasive nature and ability to provide stable fixation while preserving soft tissue integrity. However, there are contraindications, such as severely contaminated fractures, small medullary canals, and significant deformities due to prior malunions. IMN is a highly effective option for managing tibial shaft fractures, promoting optimal healing and recovery [15]. IM nails can be classified into two main categories: static and dynamic nailing. Static nailing secures the nail without allowing movement at the fracture site, making it ideal for stable fractures. In contrast, dynamic nailing allows controlled axial movement at the fracture site, promoting healing by facilitating micro-movements that stimulate callus formation. This distinction is crucial in determining the appropriate approach based on the specific characteristics of the fracture [16]. Another important classification involves reamed versus unreamed nailing techniques. Reamed nailing involves enlarging the medullary canal before inserting the nail, which can enhance stability and allow for the use of larger-diameter nails and locking screws. However, this method may risk damaging the medullary blood supply. Conversely, unreamed nailing is used when reaming poses risks, such as infection or pulmonary complications. While unreamed nails are less invasive, they tend to be weaker and are associated with higher reoperation rates due to mechanical failure [17]. IM nails can also be categorized as locking or non-locking. Locking nails use interlocking screws to secure the nail to the bone cortex, providing greater stability against rotational forces. This design is especially beneficial for unstable fractures or fractures requiring additional healing support. In contrast, non-locking nails have simpler designs without locking mechanisms, making them easier to insert but potentially less stable for complex fracture patterns [18]. IMN offers several biomechanical advantages, making it a preferred choice for fracture management. It provides strong internal fixation for early patient mobilization, leading to faster recovery. Using locking mechanisms enhances stability, reducing the risks of malrotation or collapse during healing [19]. Despite these benefits, IMN is not without complications and limitations. Infection remains a concern, particularly in cases of open fractures or poor surgical technique. Malalignment can occur if the nail is improperly positioned during surgery, potentially leading to limb deformities after healing. Additionally, soft tissue damage at the entry point may cause irritation or injury to surrounding tissues. Some patients may also experience long-term sequelae, such as persistent knee stiffness or pain, even years after surgery [20]. A comparative overview of IMN techniques for tibial shaft fractures is provided in Table 1.

**Table 1:** Comparative overview of intramedullary nailing techniques for tibial shaft fractures

Technique	Description	Indications	Advantages	Disadvantages
Standard intramedullary nailing (IMN) [21]	Involves the insertion of a metal rod into the medullary cavity of the tibia to stabilize the fracture.	Diaphyseal tibial fractures, closed or open fractures.	Minimally invasive, strong fixation, early weight-bearing.	Risk of malalignment, risk of anterior knee pain, potential for infection.
Reamed intramedullary nailing [17]	The medullary canal is reamed to a larger diameter before nail insertion.	Complex fractures, polytrauma, unstable fracture patterns.	Increased stability and higher union rates are suitable for comminuted fractures.	Increased risk of fat embolism and higher intraoperative blood loss.
Unreamed intramedullary nailing [22]	The nail is inserted without reaming the medullary canal, preserving the endosteal blood supply.	Open fractures, fractures with minimal comminution, patients with risk of fat embolism.	Reduced risk of fat embolism, less blood loss, shorter operative time.	Potential for reduced stability, higher rate of delayed union or nonunion, and potential nail breakage.
Locked intramedullary nailing [23]	Use locking screws to secure the nail proximally and distally, providing rotational and axial stability.	Segmental fractures, metaphyseal fractures, unstable fractures.	Enhanced stability, reduced risk of malrotation, suitable for complex fractures.	Increased operative time, technical difficulty, and potential for screw loosening or breakage.
Expandable intramedullary nailing [24]	Nail expands within the medullary canal to achieve fixation without the need for reaming or additional locking screws.	Diaphyseal fractures, osteoporotic bones, low-energy fractures.	Shorter operative time, less blood loss, minimally invasive.	Limited availability, potential for inadequate fixation in high-energy fractures, expensive.
Elastic intramedullary nailing (flexible nails) [25]	Use flexible nails that provide relative stability, allowing some movement at the fracture site.	Pediatric fractures, low-energy fractures, minimally displaced fractures.	Less invasive, reduced risk of soft tissue damage, suitable for growing bones.	Limited to specific fracture patterns, unsuitable for adults or complex fractures, risk of malalignment or hardware migration.

Surgical timing and fracture healing

The timing of surgical intervention is a crucial factor in fracture management, as it can significantly influence the healing process and overall patient outcomes. Early intervention, typically defined as surgery performed within 24-48 hours post-injury, aims to stabilize the fracture promptly, potentially reducing complications associated with prolonged immobilization. Conversely, delayed intervention, which occurs after 48 hours post-injury, may be required in cases of significant soft tissue swelling or when surgical resources are limited [26]. The physiological effects of surgical timing on fracture healing are profound. The inflammatory phase begins immediately following the injury, peaks within 24 hours and lasts up to seven days. Performing surgery too early can disrupt this process, potentially causing increased local tissue damage and delayed healing [12]. Surgical timing also impacts the expression of growth factors like the Vascular Endothelial Growth Factor (VEGF), which is essential for forming new blood vessels at the fracture site. Studies suggest that optimal surgery timing enhances angiogenesis, promoting better healing outcomes. Moreover, the formation of a callus, vital for bone healing, can be affected by the timing of surgery. Early intervention may interfere with natural callus formation if it occurs during the acute inflammatory phase [27]. Several clinical considerations are essential when determining the appropriate timing for surgical intervention. Patient-specific factors such as age, general health, and comorbidities are critical in recovery. Older patients or those with preexisting health conditions may benefit from delayed surgery to allow for a more comprehensive assessment of their overall condition. The nature of the fracture - whether open or closed-also significantly affects timing. Open fractures typically require urgent surgery to prevent infection, while closed fractures may permit a more measured approach [28]. Additionally, systemic factors such as the availability of surgical resources and the presence of concurrent injuries can influence the timing of surgery. In some instances, logistical constraints necessitate delaying surgery until resources are available or other injuries have been stabilized [28]. Table 2 illustrates the impact of surgical timing on fracture healing in tibial shaft injuries.

**Table 2:** Impact of surgical timing on fracture healing in tibial shaft injuries

Timing of surgery	Description	Impact on fracture healing	Advantages	Disadvantages	Recommendations
Early surgery (<24 hours) [29]	Surgical intervention within 24 hours of injury.	Promotes early stabilization and reduces inflammatory response.	Reduced risk of complications (e.g., infection, nonunion), shorter hospital stays.	Increased risk of intraoperative complications in polytrauma patients.	Preferred in stable, isolated fractures without major soft tissue injury or in hemodynamically stable patients.
Delayed surgery (24-72 hours) [30]	Surgery is performed between 24 to 72 hours post-injury.	Allows optimization of patient condition and initial swelling reduction.	A balanced approach for patient optimization and reduced complication rates.	Increased hospital stays, potential for delayed weight-bearing.	Suitable for patients requiring stabilization of other injuries or optimization of physiological status.
Late surgery (>72 hours) [31]	Surgical intervention after 72 hours of injury.	There is a higher risk of complications like infection, nonunion, and delayed union.	Allows full assessment of soft tissue injury and patient stabilization.	Increased risk of deep infection, longer rehabilitation, delayed functional recovery.	Considered when a patient’s condition contraindicates earlier surgery or in severe polytrauma cases.
Definitive early Surgery [32]	Early surgery was performed definitively with internal fixation.	Improved fracture healing and reduced complications.	Promotes early mobilization and functional recovery.	High-risk in unstable or multi-trauma patients.	Ideal for isolated fractures with good soft tissue condition.
Staged surgery [33]	Initial external fixation is followed by definitive internal fixation once the patient stabilizes (often >72 hours).	Minimizes complications by addressing soft tissue and systemic issues.	Effective in managing complex injuries and high-risk patients.	Prolonged treatment course, multiple surgeries.	Recommended in cases of severe soft tissue damage or hemodynamic instability.

Comparative analysis of early vs. delayed surgery

Literature reviews and meta-analyses have shed light on the significant impact of surgical timing on patient outcomes across a range of orthopedic conditions [34]. In elderly patients with hip fractures, a systematic review revealed that early surgery (within 24-72 hours) can reduce mortality risk by 19% [35]. In contrast, delayed surgery not only increased the one-year mortality rate by 32% but also raised the risk of pneumonia. Similarly, in cases of acute spinal cord injuries, early surgical intervention (within 24 hours) was associated with improved neurological outcomes without an increase in postoperative complications. Regarding anterior cruciate ligament (ACL) injuries, a meta-analysis showed that delaying surgery elevated the risks of meniscus tears, cartilage damage, and lower functional scores compared to early surgical treatment [36]. When comparing outcomes, early surgical interventions typically result in higher union rates and faster recovery times than delayed surgeries. For instance, IMN is associated with a significantly shorter average union time than minimally invasive plate osteosynthesis [37]. Numerous studies confirm that early surgery correlates with quicker bone healing, with tibial fractures treated through early nailing showing an average union time of approximately 18 weeks, whereas delayed approaches often prolong this period. However, early surgery may carry an increased risk of complications such as infections, particularly in cases of open fractures [37]. Early surgical intervention offers multiple advantages, including shorter hospital stays due to faster recovery and earlier mobilization, which are key to improving overall outcomes. In contrast, delayed surgery can provide more time for medical stabilization, careful surgical planning, and reduced inflammation, leading to improved surgical conditions and outcomes. Nevertheless, prolonged delays can slow the healing process and increase the likelihood of nonunion, especially in fractures where timely stabilization is essential [38].

Impact of IMN techniques on healing outcomes

Literature reviews and meta-analyses have provided valuable insights into the impact of surgical timing on patient outcomes across various orthopedic conditions. In elderly patients with hip fractures, a systematic review indicated that early surgery (within 24-72 hours) can reduce the mortality risk by 19% [39]. In contrast, delayed surgery was associated with a 32% increase in one-year mortality and a higher risk of pneumonia. Similarly, early intervention in acute spinal cord injuries (within 24 hours) significantly improved neurological outcomes without increasing the risk of postoperative complications. For ACL injuries, a meta-analysis found that delayed surgery led to higher risks of meniscus tears, cartilage damage, and lower functional scores compared to early intervention [36]. When comparing outcomes, early surgical interventions generally result in higher union rates and faster recovery times than delayed surgeries. For example, IMN is associated with a significantly shorter average union time than MIPO [37]. Studies consistently show that early surgery correlates with quicker bone healing, with tibial fractures treated through early nailing achieving an average union time of approximately 18 weeks, while delayed approaches often extend this period. However, early surgery may carry a higher risk of complications, such as infections, especially in cases of open fractures [37]. Early surgical intervention offers several benefits, including shorter hospital stays due to faster recovery and earlier mobilization, which are critical for improving overall outcomes. On the other hand, delayed surgery allows for better medical stabilization, more careful planning, and reduced inflammation, which can improve surgical conditions and outcomes. Nevertheless, prolonged delays can lead to slower healing processes and increase the risk of nonunion, particularly in fractures where timely stabilization is essential [38]. The impact of IMN techniques on healing outcomes in tibial shaft fractures is illustrated in Table 3.

**Table 3:** Impact of intramedullary nailing techniques on healing outcomes in tibial shaft fractures

Intramedullary nailing technique	Healing outcomes	Union rate	Time to union	Complications	Clinical recommendations
Standard intramedullary nailing (IMN) [40]	Provides effective stabilization and alignment.	High union rates in simple fractures.	Average time to union: 12-16 weeks.	Anterior knee pain, risk of malalignment, potential infection.	Suitable for most diaphyseal tibial fractures, especially simple, closed fractures.
Reamed intramedullary nailing [41]	Enhances stability, especially in comminuted fractures.	Higher union rates compared to unreamed nailing.	Slightly shorter time to union: 10-14 weeks.	Increased risk of fat embolism syndrome and thermal necrosis of bone.	Preferred for complex, comminuted, and polytrauma cases where higher stability is needed.
Unreamed intramedullary nailing [42]	Preserves endosteal blood supply, suitable for open fractures.	Slightly lower union rates in complex fractures.	Longer time to union: 14-18 weeks.	Higher risk of delayed union or nonunion, potential nail breakage.	Recommended for open fractures, severe soft tissue injury, and patients with contraindications to reaming.
Locked intramedullary nailing [23]	It provides additional stability with locking screws and prevents rotational and axial instability.	High union rates, especially in segmental fractures.	Similar time to union as standard IMN: 12-16 weeks.	Potential for screw loosening, breakage, and increased operative time.	Best for unstable, segmental, and metaphyseal fractures, particularly in osteoporotic bones.
Expandable intramedullary nailing [43]	Minimally invasive technique with rapid expansion to achieve fixation.	Comparable union rates to standard nailing in selected cases.	Time to union varies depending on the fracture pattern.	Inadequate fixation in high-energy fractures, higher cost, limited availability.	Suitable for low-energy fractures in osteoporotic bone or patients with contraindications to traditional nailing techniques.
Elastic intramedullary nailing [44]	Allows micro-movement at the fracture site, promoting callus formation in pediatric fractures.	High union rates in pediatric fractures.	Rapid healing: 8-12 weeks.	Limited application in adults, risk of malalignment or hardware migration.	Recommended for pediatric patients and simple diaphyseal fractures with low energy in young adults.

Complications associated with surgical timing and technique

The timing of surgical intervention and the choice of technique in treating tibial shaft fractures play a critical role in influencing the risk of complications, including infection, nonunion, malunion, soft tissue damage, and implant failure [45]. Research indicates that delayed surgical intervention is associated with increased infection rates. Specifically, a longer interval between diagnosis and surgery correlates with a higher risk of major surgical complications, such as infections [46]. Early intervention, typically performed within 24 to 72 hours of injury, has significantly reduced the risk of wound infections compared to delayed procedures. Additionally, the surgical technique used also impacts infection rates. IMN, for example, is associated with lower infection rates than other methods like MIPO, which has demonstrated higher infection rates in certain studies [46]. Surgical timing also significantly impacts the rates of nonunion and malunion in tibial shaft fractures. Early surgical intervention is linked to lower rates of these complications, while delayed surgery complicates the healing process, increasing the likelihood of nonunion or malalignment. A systematic review showed that early IMN reduced infection risks and improved union rates compared to delayed nailing. Furthermore, the surgical technique can mitigate these risks; the use of blocking screws during IMN enhances fracture stability and reduces the likelihood of nonunion and malalignment by providing better structural support during the healing process [47]. Other complications associated with both surgical timing and technique include soft tissue damage, implant failure, and compartment syndrome. Delayed surgery can exacerbate soft tissue injuries, increasing the risk of complications such as compartment syndrome. Early intervention tends to minimize soft tissue damage by reducing swelling and achieving better fracture alignment during the initial treatment [48]. Surgical timing also influences implant failure, as delayed surgeries can place additional mechanical stress on implants due to poor alignment or inadequate stabilization during healing. Furthermore, compartment syndrome, a serious complication, is more likely to develop when there is a significant delay in addressing tibial fractures, particularly when swelling occurs before surgery [48]. Immediate surgical intervention helps to alleviate pressure and reduce the risk of compartment syndrome. Table 4 summarizes the complications associated with surgical timing and IMN techniques for tibial shaft fractures.

**Table 4:** Complications associated with surgical timing and intramedullary nailing techniques for tibial shaft fractures

Category	Complication	Description	Associated surgical timing	Associated nailing technique	Prevention/management
Infection [49]	Superficial or deep infection	Infection at the surgical site or within the bone (osteomyelitis).	Late surgery (>72 hours), open fractures	Reamed nailing, standard IMN	Early debridement, prophylactic antibiotics, staged surgery in severe cases.
Nonunion [50]	Delayed or absence of fracture healing	Failure of the fracture to heal within the expected timeframe.	Early (<24 hours) or late (>72 hours)	Unreamed nailing, standard IMN	Optimal surgical timing, appropriate technique selection, use of bone graft or BMPs.
Malalignment [51]	Angular, rotational, or shortening deformities	Incorrect alignment of the fractured bone segments.	Early surgery (<24 hours)	Unlocked or standard IMN	Use of locked nailing, intraoperative imaging, and precise surgical technique.
Fat embolism syndrome [52]	Fat emboli entering the bloodstream	Characterized by respiratory distress, cerebral symptoms, and petechial rash.	Early surgery (<24 hours)	Reamed nailing	Use unreamed nailing in high-risk patients and monitor oxygenation and hemodynamics.
Compartment syndrome [53]	Increased pressure within a muscle compartment	This leads to reduced blood flow and tissue damage and requires emergency intervention.	Early (<24 hours) or delayed (24-72 hours)	Any technique	Early diagnosis, fasciotomy, avoid tight cast or splinting, monitor limb perfusion postoperatively.
Anterior knee pain [54]	Pain in the anterior knee, particularly in the patellar region	Commonly associated with nail insertion point, affecting long-term functional outcomes.	Any timing	Standard IMN, locked nailing	Use proper entry points, consider a semi-extended position for nail insertion, and avoid prominent nail heads.
Delayed union [55]	Prolonged time for fracture healing	Fracture takes longer than expected to heal but eventually unite.	Delayed (24-72 hours), late (>72 hours)	Unreamed nailing	Optimize patient health, monitor fracture healing with radiographs, and consider bone stimulators if needed.
Hardware failure [56]	Nail or screw breakage	Breakage or loosening of intramedullary nails or locking screws due to mechanical stress or poor healing.	Late surgery (>72 hours)	Unreamed nailing, elastic nailing	Proper technique, appropriate nail size, adequate locking, follow-up for early detection, and timely surgical intervention.
Thermal necrosis [57]	Bone damage due to heat generated during reaming	It can compromise bone healing and lead to nonunion or infection.	Early surgery (<24 hours)	Reamed nailing	Use low-speed and intermittent reaming with cooling and consider the unreamed technique in high-risk cases.
Soft tissue injury [58]	Damage to muscles, tendons, or nerves	Iatrogenic injury during nail insertion or due to prolonged surgery duration.	Early (<24 hours) or late (>72 hours)	Any technique	Minimize soft tissue handling, use meticulous surgical technique, and ensure proper exposure and retraction.

Guidelines for optimal surgical timing and technique selection

Evidence-based guidelines emphasize the benefits of early surgical intervention - preferably within 24 to 72 hours - for tibial shaft fractures. Early stabilization is linked to reduced complications, shorter hospital stays, and improved functional recovery. This prompt approach minimizes soft tissue complications and facilitates quicker rehabilitation. However, surgical timing may vary depending on the fracture type. For instance, complex fractures or those accompanied by significant soft tissue injury may require a delayed approach to allow for swelling reduction and patient stabilization before surgery [59]. Patient-specific factors such as age, overall health, and activity level are also critical in determining the optimal timing for surgery. Elderly patients or those with significant comorbidities may necessitate a more cautious approach, delaying surgery until their health status is carefully evaluated [59]. The choice of IMN technique is another crucial factor in optimizing outcomes for tibial shaft fractures. Deciding between reamed and unreamed nails depends largely on the fracture's characteristics and location. Reamed nails offer enhanced stability but may disrupt blood flow to the fracture site, which can be a disadvantage in certain cases. In contrast, unreamed nails are often preferred for less severe fractures or when minimizing infection risk is a priority, such as in open fractures [60]. Surgical approach selection is equally important; the suprapatellar approach is gaining popularity over the traditional infrapatellar technique because it allows for better fracture reduction control, reduces radiation exposure during fluoroscopy, and improves alignment during nail insertion. For fractures in patients with compromised bone quality, such as osteoporosis, unreamed nails may be the better option due to their flexibility and lower risk of complications from reaming [60]. A structured decision-making algorithm is useful for orthopedic surgeons in determining the appropriate timing and technique for surgery. The process starts with evaluating the fracture type - whether simple or complex, open, or closed. Following this, patient factors such as age, comorbidities, and activity level are considered. Early nailing within 24 to 72 hours is typically recommended for simple fractures. In contrast, complex fractures with significant soft tissue damage may require temporary external fixation before definitive nailing can occur [61]. Once the surgical timing is decided, the next step involves selecting the appropriate nailing technique. For mid-diaphyseal fractures, the decision between reamed or unreamed nails is based on bone quality. The suprapatellar approach is generally favored for distal tibial fractures due to its advantages in achieving better alignment and reducing operative time. Postoperative management should closely monitor potential complications such as infection or malalignment while implementing a rehabilitation plan tailored to the patient’s recovery [62]. Adhering to these guidelines and utilizing a structured decision-making approach, orthopedic surgeons can make informed decisions regarding surgical timing and technique selection, ultimately optimizing outcomes for patients with tibial shaft fractures [63]. Table 5 outlines the guidelines for optimal surgical timing and technique selection in tibial shaft fractures.

**Table 5:** Guidelines for optimal surgical timing and technique selection for tibial shaft fractures

Clinical scenario	Optimal surgical timing	Recommended technique	Rationale	Considerations
Isolated closed tibial shaft fracture [64]	Early surgery (<24 hours)	Standard intramedullary nailing (IMN)	Early stabilization promotes faster recovery and reduced complications.	Ensure patient hemodynamic stability and adequate preoperative imaging for planning.
Polytrauma with tibial fracture [65]	Delayed surgery (24-72 hours)	Reamed locked intramedullary nailing	Allows time for patient stabilization and comprehensive assessment.	Prioritize life-threatening injuries first; optimize patient’s physiological status before surgery.
Open fracture (Grade I & II) [66]	Early surgery (<24 hours)	Unreamed intramedullary nailing	Reduces risk of infection, provides early stability.	Adequate debridement is crucial; ensure proper wound management and use of prophylactic antibiotics.
Open fracture (Grade III) [66]	Staged surgery	Initial external fixation, followed by reamed locked IMN	Manages soft tissue and systemic issues before definitive fixation.	Early wound coverage and soft tissue management are critical; delay definitive surgery until soft tissue healing.
Severe soft tissue injury [67]	Late surgery (>72 hours)	Staged approach (external fixation then IMN)	Minimizes risk of infection and wound complications.	Requires careful monitoring and planning; consider flap coverage if needed.
Pediatric tibial shaft fracture [68]	Early surgery (<24 hours)	Elastic intramedullary nailing	Minimally invasive, preserves growth plate and blood supply.	Ensure proper fracture alignment and stability; avoid growth plate injury.
Metaphyseal or segmental fracture [69]	Early surgery (<24 hours)	Reamed locked intramedullary nailing	Provides better stability and alignment.	Use locking screws to prevent rotational or axial instability; consider plate augmentation if needed.
Severe osteoporotic bone [70]	Delayed surgery (24-72 hours)	Expandable intramedullary nailing	Provides adequate fixation in fragile bone with minimal invasion.	Monitor bone quality intraoperatively; consider bone graft or augmentation for additional support.
Comminuted or highly unstable fracture [71]	Early surgery (<24 hours)	Reamed locked intramedullary nailing	Ensures optimal stability and alignment.	Use larger diameter nail for increased strength; consider adjunctive fixation methods if necessary.
High-risk patients (e.g., coagulopathy, severe COPD) [72]	Delayed surgery (24-72 hours)	Unreamed intramedullary nailing	Minimizes intraoperative stress and risk.	Close monitoring of patient condition; ensure perioperative optimization and use of prophylactic measures.

Future directions and research gaps

Recent advancements in IMN techniques for tibial shaft fractures have focused on enhancing patient recovery and improving surgical outcomes. Among these innovations, the suprapatellar nailing approach has gained popularity due to its potential benefits over the traditional infrapatellar method. Studies suggest that this approach reduces operative times, reduces radiation exposure, and improves functional recovery. Additionally, it is associated with fewer complications such as malunion and infection rates, making it a preferred method in certain clinical scenarios [73]. Another promising development is the use of poller-blocking screws, which have proven effective in preventing malalignment and nonunion, particularly in distal tibial fractures. While these screws improve fracture stability, they may increase surgical time and radiation exposure due to the need for precise placement. In parallel, advancements in MIPO are being explored as alternatives to IMN, particularly in patients with poor soft tissue conditions. These minimally invasive techniques aim to reduce complications related to more traditional open surgeries and are valuable options in challenging cases [73]. However, several gaps remain in the current literature concerning the long-term efficacy of these newer techniques. There is a need for extensive longitudinal studies to assess long-term functional recovery and complications, which would provide a clearer picture of the relative benefits of different approaches over time [74]. In addition, comparative studies between the suprapatellar and infrapatellar approaches and between IMN and MIPO are required to define the most effective treatment protocols for various fracture types. Standardizing these protocols, especially in low-resource settings, is critical to ensure global consistency in care, as current practices vary significantly across regions based on available resources [74]. The future of tibial shaft fracture treatment will likely focus more on personalized medicine, with treatment decisions tailored to each patient's unique profile. Surgical timing and techniques could be customized based on age, comorbidities, and specific fracture characteristics, enhancing treatment outcomes. For instance, while early surgical intervention may benefit certain patients, others might require a more conservative approach due to soft tissue damage or other medical concerns. Moreover, personalizing surgical techniques based on anatomical variations or previous injuries may lead to more favorable outcomes by aligning the surgical plan with the patient’s specific needs [75]. Integrating advanced imaging techniques and computer-assisted navigation into surgery also promises to enhance precision in treating tibial shaft fractures, allowing surgeons to tailor interventions further to each patient's anatomy [75]. Table 6 outlines the future directions and research gaps in IMN and surgical timing for tibial shaft fractures.

**Table 6:** Future directions and research gaps in intramedullary nailing and surgical timing for tibial shaft fractures

Area of focus	Future directions	Research gaps	Potential impact
Optimal surgical timing [76]	Investigate specific patient and injury factors influencing ideal surgical timing.	Limited high-quality randomized controlled trials (RCTs) on timing impact, especially in polytrauma and open fractures.	Improved patient outcomes and tailored surgical timing strategies.
Advanced imaging techniques [77]	Utilize advanced imaging (e.g., 3D CT, MRI) for better preoperative planning and postoperative assessment.	Lack of standardized protocols for imaging in complex fracture patterns and soft tissue assessment.	Enhanced preoperative planning and better prediction of healing outcomes.
Biomechanical studies on nailing techniques [78]	Develop new intramedullary nails with improved biomechanical properties (e.g., expandable, bioabsorbable nails).	Insufficient data on the long-term performance and complications of new nail designs.	Improved fixation stability, reduced complications, and faster healing.
Minimally invasive surgical techniques [79]	Research on less invasive approaches to minimize soft tissue damage and promote early recovery.	Limited studies comparing minimally invasive vs. standard approaches in diverse patient populations.	Reduced surgical morbidity, faster recovery times, and better functional outcomes.
Patient-specific factors [80]	Studies on how age, bone quality, comorbidities, and fracture characteristics influence technique selection.	Lack of large-scale, multicenter studies addressing patient-specific outcomes.	Personalized treatment plans, optimized outcomes for diverse patient groups.
Enhanced recovery protocols [81]	Develop and evaluate enhanced recovery protocols specific to tibial fractures (e.g., pain management, rehabilitation).	Few studies integrate multidisciplinary recovery approaches (e.g., physiotherapy, pain management, nutritional support).	Shorter hospital stays, faster return to function, improved quality of life.
Augmentation techniques [82]	Research using bone grafts, growth factors, and other augmentation techniques in nonunion cases.	Limited high-quality evidence on the efficacy and safety of various augmentation methods in intramedullary nailing.	Increased union rates, reduced time to union, lower reoperation rates.
Complication management strategies [83]	Develop standardized guidelines for preventing and managing complications like infection and nonunion.	Inconsistent management strategies across different centers; lack of consensus on best practices.	Lower complication rates, standardized care, better long-term outcomes.
Long-term functional outcomes [84]	Investigate the impact of surgical timing and technique on long-term functional outcomes (e.g., mobility, pain).	Limited long-term follow-up studies focusing on functional and quality-of-life outcomes.	Better understanding of patient satisfaction and functional recovery.
Cost-effectiveness analysis [85]	Analyze the cost-effectiveness of different surgical timings and techniques in various healthcare settings.	Few studies evaluating the economic impact of different treatment strategies in low-resource settings.	Optimized resource allocation, cost-effective treatment planning.

## Conclusions

In conclusion, the timing of surgical intervention and the choice of IMN technique play pivotal roles in determining the outcomes of tibial shaft fracture healing. Early surgical intervention offers the potential for reduced hospital stays, quicker mobilization, and prevention of secondary complications; however, it may also increase the risk of infection and soft tissue damage, particularly in cases of open fractures. Conversely, delayed surgery allows for better preoperative preparation and soft tissue recovery but may result in delayed healing and higher nonunion rates. IMN has proven to be the gold standard for stabilizing tibial shaft fractures due to its biomechanical superiority and minimally invasive nature. Yet, the debate over the optimal nailing technique - whether reamed versus unreamed or static versus dynamic - remains, with each approach presenting unique benefits and challenges. This review underscores the importance of individualized patient care, considering the fracture characteristics, patient comorbidities, and the clinical context to optimize surgical timing and technique. Ultimately, evidence-based guidelines integrating these variables are crucial to improving patient outcomes, minimizing complications, and enhancing the functional recovery of individuals with tibial shaft fractures.

## References

[1] Tamburini L, Zeng F, Neumann D (2023). A review of tibial shaft fracture fixation methods. Trauma Care.

[2] Denisiuk M, Afsari A (2024). Femoral Shaft Fractures. https://www.ncbi.nlm.nih.gov/books/NBK556057/.

[3] Mundi R, Axelrod D, Heels-Ansdell D (2020). Nonunion in patients with tibial shaft fractures: is early physical status associated with fracture healing?. Cureus.

[4] Houben IB, Raaben M, Van Basten Batenburg M, Blokhuis TJ (2018). Delay in weight bearing in surgically treated tibial shaft fractures is associated with impaired healing: a cohort analysis of 166 tibial fractures. Eur J Orthop Surg Traumatol.

[5] Singaram S, Naidoo M (2019). The physical, psychological and social impact of long bone fractures on adults: a review. Afr J Prim Health Care Fam Med.

[6] Duyos OA, Beaton-Comulada D, Davila-Parrilla A, Perez-Lopez JC, Ortiz K, Foy-Parrilla C, Lopez-Gonzalez F (2017). Management of open tibial shaft fractures: does the timing of surgery affect outcomes?. J Am Acad Orthop Surg.

[7] Marais LC, Zalavras CG, Moriarty FT, Kühl R, Metsemakers WJ, Morgenstern M (2024). The surgical management of fracture-related infection. Surgical strategy selection and the need for early surgical intervention. J Orthop.

[8] Radaideh A, Alrawashdeh MA, Al Khateeb AH (2022). Outcomes of treating tibial shaft fractures using intramedullary nailing (imn) versus minimally invasive percutaneous plate osteosynthesis (MIPPO). Med Arch.

[9] Aggarwal S, Rajnish RK, Kumar P, Srivastava A, Rathor K, Haq RU (2023). Comparison of outcomes of retrograde intramedullary nailing versus locking plate fixation in distal femur fractures: a systematic review and meta-analysis of 936 patients in 16 studies. J Orthop.

[10] Bourne M, Sinkler MA, Murphy PB (2024). Anatomy, Bony Pelvis and Lower Limb: Tibia. https://www.ncbi.nlm.nih.gov/books/NBK526053/.

[11] Almansour H, Jacoby J, Baumgartner H, Reumann MK, Nikolaou K, Springer F (2020). Injury of the tibial nutrient artery canal during external fixation for lower extremity fractures: a computed tomography study. J Clin Med.

[12] Sheen JR, Mabrouk A, Garla VV (2024). Fracture Healing Overview. https://www.ncbi.nlm.nih.gov/books/NBK551678/.

[13] Glatt V, Evans CH, Tetsworth K (2016). A concert between biology and biomechanics: the influence of the mechanical environment on bone healing. Front Physiol.

[14] Camargo FP, Gaiarsa GP, Camargo OP, Reis PR, Silva JD, Kojima KE (2021). The missing link in the history of the locked intramedullary nail. Acta Ortop Bras.

[15] Metcalf KB, Brown CC, Barksdale EM 3rd, Wetzel RJ, Sontich JK, Ochenjele G (2020). Clinical outcomes after intramedullary nailing of intraarticular distal tibial fractures: a retrospective review. J Am Acad Orthop Surg Glob Res Rev.

[16] Omerovic D, Lazovic F, Hadzimehmedagic A (2015). Static or dynamic intramedullary nailing of femur and tibia. Med Arch.

[17] Li AB, Zhang WJ, Guo WJ, Wang XH, Jin HM, Zhao YM (2016). Reamed versus unreamed intramedullary nailing for the treatment of femoral fractures: a meta-analysis of prospective randomized controlled trials. Medicine (Baltimore).

[18] (2024). Intramedullary nailing for Simple fracture, oblique. https://surgeryreference.aofoundation.org/orthopedic-trauma/adult-trauma/tibial-shaft/simple-fracture-oblique/intramedullary-nailing.

[19] Bong MR, Kummer FJ, Koval KJ, Egol KA (2007). Intramedullary nailing of the lower extremity: biomechanics and biology. J Am Acad Orthop Surg.

[20] Makridis KG, Tosounidis T, Giannoudis PV (2013). Management of infection after intramedullary nailing of long bone fractures: treatment protocols and outcomes. Open Orthop J.

[21] Cereijo C, Attum B, Rodriguez-Buitrago A, Jahangir AA, Obremskey W (2018). Intramedullary nail fixation of tibial shaft fractures: suprapatellar approach. JBJS Essent Surg Tech.

[22] Högel F, Gerlach UV, Südkamp NP, Müller CA (2010). Pulmonary fat embolism after reamed and unreamed nailing of femoral fractures. Injury.

[23] Li YH, Yu T, Shao W, Liu Y, Zhu D, Tan L (2020). Distal locked versus unlocked intramedullary nailing for stable intertrochanteric fractures, a systematic review and meta-analysis. BMC Musculoskelet Disord.

[24] Akdemir M, Biçen Ç, Özkan M, Ekin A (2021). Comparison of expandable and locked intramedullary nailing for humeral shaft fractures. Cureus.

[25] Vasilescu DE, Cosma D (2014). Elastic stable intramedullary nailing for fractures in children - principles, indications, surgical technique. Clujul Med.

[26] Dong LQ, Yin H, Wang CX, Hu WF (2014). Effect of the timing of surgery on the fracture healing process and the expression levels of vascular endothelial growth factor and bone morphogenetic protein-2. Exp Ther Med.

[27] Johnson KE, Wilgus TA (2014). Vascular endothelial growth factor and angiogenesis in the regulation of cutaneous wound repair. Adv Wound Care (New Rochelle).

[28] Schmidt AP, Stefani LC (2022). How to identify a high-risk surgical patient?. Braz J Anesthesiol.

[29] Volpin G, Pfeifer R, Saveski J, Hasani I, Cohen M, Pape HC (2020). Damage control orthopaedics in polytraumatized patients- current concepts. J Clin Orthop Trauma.

[30] Liu S, Qiang L, Yang Q (2023). Delayed surgery is associated with adverse outcomes in patients with hip fracture undergoing hip arthroplasty. BMC Musculoskelet Disord.

[31] Nicolaides M, Vris A, Heidari N, Bates P, Pafitanis G (2021). The effect of delayed surgical debridement in the management of open tibial fractures: a systematic review and meta-analysis. Diagnostics (Basel).

[32] Nahm NJ, Como JJ, Wilber JH, Vallier HA (2011). Early appropriate care: definitive stabilization of femoral fractures within 24 hours of injury is safe in most patients with multiple injuries. J Trauma.

[33] Kornah BA, Abdelaziz M, I Abulsoud M, Abdel Ghani T, Seleem N, Alshal EA, Abdel-AAl MA (2021). Management of failed external fixation by two‐staged internal osteosynthesis in the lower limb. Orthop Surg.

[34] Bhandari M, Devereaux PJ, Montori V, Cinà C, Tandan V, Guyatt GH (2004). Users' guide to the surgical literature: how to use a systematic literature review and meta-analysis. Can J Surg.

[35] Klestil T, Röder C, Stotter C (2017). Immediate versus delayed surgery for hip fractures in the elderly patients: a protocol for a systematic review and meta-analysis. Syst Rev.

[36] Xue F, Zhan SZ, Zhang DY, Jiang BG (2021). Early versus delayed surgery for acute traumatic cervical/thoracic spinal cord injury in beijing, china: the results of a prospective, multicenter nonrandomized controlled trial. Orthop Surg.

[37] Elnewishy A, Elkholy M, Hamada A, Salem M (2023). Comparing minimally invasive percutaneous plate osteosynthesis with interlocking intramedullary nail fixation for the management of adult extra-articular distal tibial fractures: a comprehensive systematic review and meta-analysis. Cureus.

[38] Tazreean R, Nelson G, Twomey R (2022). Early mobilization in enhanced recovery after surgery pathways: current evidence and recent advancements. J Comp Eff Res.

[39] Klestil T, Röder C, Stotter C (2018). Impact of timing of surgery in elderly hip fracture patients: a systematic review and meta-analysis. Sci Rep.

[40] Li X, Chen K, Xue H, Cheng J, Yu X (2024). Efficacy comparison between intramedullary nail fixation and plate fixation in distal tibia fractures: a meta-analysis of randomized controlled trials. J Orthop Surg Res.

[41] Choudary D, Kanthimathi B (2012). A prospective comparative study of reamed vs. unreamed nailing in fractures shaft of tibia. Malays Orthop J.

[42] Babalola OM, Ibraheem GH, Ahmed BA, Olawepo A, Agaja SB, Adeniyi A (2016). Open intramedullary nailing for segmental long bone fractures: an effective alternative in a resource-restricted environment. Niger J Surg.

[43] Rose DM, Smith TO, Nielsen D, Hing CB (2013). Expandable intramedullary nails in lower limb trauma: a systematic review of clinical and radiological outcomes. Strategies Trauma Limb Reconstr.

[44] Kang SN, Mangwani J, Ramachandran M, Paterson JM, Barry M (2011). Elastic intramedullary nailing of paediatric fractures of the forearm: a decade of experience in a teaching hospital in the United Kingdom. J Bone Joint Surg Br.

[45] Gálvez-Sirvent E, Ibarzábal-Gil A, Rodríguez-Merchán EC (2022). Complications of the surgical treatment of fractures of the tibial plateau: prevalence, causes, and management. EFORT Open Rev.

[46] Cheng H, Chen BP, Soleas IM, Ferko NC, Cameron CG, Hinoul P (2017). Prolonged operative duration increases risk of surgical site infections: a systematic review. Surg Infect (Larchmt).

[47] Albright PD, Ali SH, Jackson H, Haonga BT, Eliezer EN, Morshed S, Shearer DW (2020). Delays to surgery and coronal malalignment are associated with reoperation after open tibia fractures in Tanzania. Clin Orthop Relat Res.

[48] Igoumenou VG, Kokkalis ZT, Mavrogenis AF (2019). Fasciotomy Wound Management.

[49] Momodu II, Savaliya V (2024). Osteomyelitis. https://www.ncbi.nlm.nih.gov/books/NBK532250/.

[50] Thomas JD, Kehoe JL (2024). Bone Nonunion. https://www.ncbi.nlm.nih.gov/books/NBK554385/.

[51] Branca Vergano L, Coviello G, Monesi M (2020). Rotational malalignment in femoral nailing: prevention, diagnosis and surgical correction. Acta Biomed.

[52] Adeyinka A, Pierre L (2024). Fat Embolism. https://www.ncbi.nlm.nih.gov/books/NBK499885/.

[53] Torlincasi AM, Lopez RA, Waseem M (2024). Acute Compartment Syndrome. https://www.ncbi.nlm.nih.gov/books/NBK448124/.

[54] Gencer B, Yiğit A, Çamoğlu C, Çulcu A, Dogan O (2023). Can anterior knee pain be explained by patella position after infrapatellar tibia intramedullary nailing?. Cureus.

[55] Miclau T, Lu C, Thompson Z, Choi P, Puttlitz C, Marcucio R, Helms JA (2007). Effects of delayed stabilization on fracture healing. J Orthop Res.

[56] Bäcker HC, Heyland M, Wu CH, Perka C, Stöckle U, Braun KF (2022). Breakage of intramedullary femoral nailing or femoral plating: how to prevent implant failure. Eur J Med Res.

[57] Timon C, Keady C (2019). Thermal osteonecrosis caused by bone drilling in orthopedic surgery: a literature review. Cureus.

[58] Dora C, Leunig M, Beck M, Rothenfluh D, Ganz R (2001). Entry point soft tissue damage in antegrade femoral nailing: a cadaver study. J Orthop Trauma.

[59] Whiting PS, Obremskey W, Johal H, Shearer D, Volgas D, Balogh ZJ (2024). Open fractures: evidence-based best practices. OTA Int.

[60] Xue D, Zheng Q, Li H, Qian S, Zhang B, Pan Z (2010). Reamed and unreamed intramedullary nailing for the treatment of open and closed tibial fractures: a subgroup analysis of randomised trials. Int Orthop.

[61] Solyom A, Moldovan F, Moldovan L, Strnad G, Fodor P (2024). Clinical workflow algorithm for preoperative planning, reduction and stabilization of complex acetabular fractures with the support of three-dimensional technologies. J Clin Med.

[62] Bhandari M, Guyatt G, Tornetta P 3rd, Schemitsch EH, Swiontkowski M, Sanders D, Walter SD (2008). Randomized trial of reamed and unreamed intramedullary nailing of tibial shaft fractures. J Bone Joint Surg Am.

[63] Metsemakers WJ, Kortram K, Ferreira N (2021). Fracture-related outcome study for operatively treated tibia shaft fractures (F.R.O.S.T.): registry rationale and design. BMC Musculoskelet Disord.

[64] Auston DA, Meiss J, Serrano R (2017). Percutaneous or open reduction of closed tibial shaft fractures during intramedullary nailing does not increase wound complications, infection or nonunion rates. J Orthop Trauma.

[65] Yokoyama K, Itoman M, Uchino M, Fukushima K, Nitta H, Kojima Y (2008). Immediate versus delayed intramedullary nailing for open fractures of the tibial shaft: a multivariate analysis of factors affecting deep infection and fracture healing. Indian J Orthop.

[66] Sop JL, Sop A (2024). Open Fracture Management. https://www.ncbi.nlm.nih.gov/books/NBK448083/.

[67] Patil V, Sarkar B, Mir MA (2022). Immediate versus late flap coverage for traumatic soft tissue defects of lower extremity: a comparative observational study. Cureus.

[68] Pogorelić Z, Vegan V, Jukić M, Llorente Muñoz CM, Furlan D (2022). Elastic stable intramedullary nailing for treatment of pediatric tibial fractures: a 20-year single center experience of 132 cases. Children (Basel).

[69] Zelle BA, Boni G (2015). Safe surgical technique: intramedullary nail fixation of tibial shaft fractures. Patient Saf Surg.

[70] Elbarbary AN, Hassen S, Badr IT (2021). Outcome of intramedullary nail for fixation of osteoporotic femoral shaft fractures in the elderly above 60. Injury.

[71] Kempf I, Grosse A, Beck G (1985). Closed locked intramedullary nailing. Its application to comminuted fractures of the femur. J Bone Joint Surg Am.

[72] Licker M, Schweizer A, Ellenberger C, Tschopp JM, Diaper J, Clergue F (2007). Perioperative medical management of patients with COPD. Int J Chron Obstruct Pulmon Dis.

[73] Ringenberg JD, Tobey JL, Horinek JL, Teague DC (2022). Suprapatellar versus infrapatellar approach for intramedullary nail fixation of tibial shaft fractures: a review of the literature. OTA Int.

[74] Sagar B V S, Nandi SS, Kulkarni SR, Bagewadi R (2023). Functional outcomes of tibia fractures treated with intramedullary interlocking nails by suprapatellar approach: a prospective study. Cureus.

[75] Swart E, Lasceski C, Latario L, Jo J, Nguyen US (2020). Modern treatment of tibial shaft fractures: is there a role today for closed treatment?. Injury.

[76] You DZ, Schneider PS (2020). Surgical timing for open fractures: middle of the night or the light of day, which fractures, what time?. OTA Int.

[77] Voss EE, Goode RD, Cook JL, Crist BD (2022). Survey of orthopaedic trauma providers: is mri superior to ct scan for evaluating and preoperative planning for tibial plateau fractures?. Mo Med.

[78] Johnson CW, Carmichael KD, Morris RP, Gilmer B (2009). Biomechanical study of flexible intramedullary nails. J Pediatr Orthop.

[79] Darzi SA, Munz Y (2004). The impact of minimally invasive surgical techniques. Annu Rev Med.

[80] Tiihonen R, Helkamaa T, Nurmi-Lüthje I, Kaukonen JP, Kataja M, Lüthje P (2022). Patient-specific factors affecting survival following hip fractures-a 14-year follow-up study in Finland. Arch Osteoporos.

[81] Kowa CY, Jin Z, Gan TJ (2022). Framework, component, and implementation of enhanced recovery pathways. J Anesth.

[82] Mohamed MA, Noaman HH, Soroor YO, Elsayed M (2022). Plate augmentation and bone grafting in treatment of femoral shaft nonunion initially fixed by intramedullary nail. SICOT J.

[83] Olansen J, Ibrahim Z, Aaron RK (2024). Management of garden-i and ii femoral neck fractures: perspectives on primary arthroplasty. Orthop Res Rev.

[84] Hoag CC, Gotto GT, Morrison KB, Coleman GU, Macneily AE (2008). Long-term functional outcome and satisfaction of patients with hypospadias repaired in childhood. Can Urol Assoc J.

[85] Jamison DT, Breman JG, Measham AR (2006). Priorities in Health. World Bank.

